# Influence of SARS-CoV-2 surveillance outputs produced by the UK health security agency (UKHSA) outbreak surveillance team on decision-making by local stakeholders

**DOI:** 10.1186/s12889-023-15784-8

**Published:** 2023-05-22

**Authors:** Katriina Willgert, Jo Hardstaff, Stephanie Shadwell, Alex Bhattacharya, Paula Blomquist, Roberto Vivancos, Ian Simms

**Affiliations:** 1 Outbreak Surveillance Team, Field Services, UK Health Security Agency, London, UK; 2 Disease Dynamics Unit, Department of Veterinary Medicine, University of Cambridge, Madingley Road, CB3 0ES Cambridge, UK; 3 Data Product Development, Data Operations, UK Health Security Agency, London, UK

**Keywords:** Surveillance methods, Surveillance reports, Epidemiological intelligence, Evaluation, Outbreak response, Pandemic preparedness, SARS-CoV-2, COVID-19

## Abstract

**Background:**

The UK Health Security Agency (UKHSA) COVID-19 Outbreak Surveillance Team (OST) was established in June 2020 to provide Local Authorities (LAs) in England with surveillance intelligence to aid their response to the SARS-CoV-2 epidemic. Reports were produced using standardised metrics in an automated format. Here we evaluate how the SARS-CoV-2 surveillance reports influenced decision making, how resources evolved and how they could be refined to meet the requirements of stakeholders in the future.

**Methods:**

Public health professionals (n = 2,400) involved in the COVID-19 response from the 316 English LAs were invited to take part in an online survey. The questionnaire covered five themes: (i) report use; (ii) influence of surveillance outputs on local intervention strategies; (iii) timeliness; (iv) current and future data requirements; and (v) content development.

**Results:**

Of the 366 respondents to the survey, most worked in public health, data science, epidemiology, or business intelligence. Over 70% of respondents used the LA Report and Regional Situational Awareness Report daily or weekly. The information had been used by 88% to inform decision making within their organisations and 68% considered that intervention strategies had been instituted as a result of these decisions. Examples of changes instigated included targeted communications, pharmaceutical and non-pharmaceutical interventions, and the timing of interventions. Most responders considered that the surveillance content had reacted well to evolving demands. The majority (89%) said that their information requirements would be met if the surveillance reports were incorporated into the COVID-19 Situational Awareness Explorer Portal. Additional information suggested by stakeholders included vaccination and hospitalisation data as well as information on underlying health conditions, infection during pregnancy, school absence and wastewater testing.

**Conclusions:**

The OST surveillance reports were a valuable information resource used by local stakeholders in their response to the SARS-CoV-2 epidemic. Control measures that affect disease epidemiology and monitoring requirements need to be considered in the continuous maintenance of surveillance outputs. We identified areas for further development and, since the evaluation, information on repeat infections and vaccination data have been included in the surveillance reports. Furthermore, timeliness of publications has been improved by updating the data flow pathways.

**Supplementary Information:**

The online version contains supplementary material available at 10.1186/s12889-023-15784-8.

## Background

Severe acute respiratory syndrome coronavirus 2 (SARS-CoV-2) was first recorded in the United Kingdom (UK) in late January 2020 [1]. In June 2020, the UK Health Security Agency (UKHSA, formerly Public Health England) COVID-19 Outbreak Surveillance Team (OST) was created to act as the central information point for the production of timely, accurate and detailed surveillance reports to support the work of health specialists in each of the 316 English Lower Tier Local Authorities (LA; average total population = 360,000 people). Several different reports were produced using data collected through Covid-19 surveillance systems for a variety of specialist audiences (supplementary material, Table S1 and S2). The suite of data outputs included in the reports produced by OST used information from six UKHSA SARS-CoV-2 surveillance datasets (HPZone, Unified Sample Database (USD), EpiCell data, Second Generation Surveillance System (SGSS), Severe Acute Respiratory Infection (SARI) watch and Syndromic data), population denominators and mortality data from the Office for National Statistics (ONS), and mortality, hospital bed and mechanical ventilation records from the National Health Service (NHS). Production was automated by using R software for statistical computing and graphics [2], and the reports were distributed through a web-based document management and storage system (Microsoft SharePoint). The centralised and automated generation of the surveillance reports allowed for effective production and distribution of information to stakeholders and the rapid introduction of episode reporting. A Methods Companion document was produced for stakeholders which included technical details of the metrics and data sources used. Metrics were agreed across UKHSA by the Incident Metrics Co-ordination Group in line with those specified for UKHSA publications and reports [3], which enabled comparisons to be made between geographical regions over space and time.

The UKHSA COVID-19 Situational Awareness Explorer Portal, which included interactive data presentations and underlying databases, was launched in July 2020 and could be accessed by public health professionals in local government. This allowed greater flexibility in the way that LAs could develop their own in-house epidemiological intelligence reports alongside those produced by OST.

Ensuring that surveillance outputs anticipate and adapt to the changing requirements of stakeholders is crucial to maintaining effective prevention and control strategies. The content of the reports was curated by OST’s health protection specialists. Modifications were made in response to changes in prevention and control priorities identified by the national incident management team (IMT), the content of surveillance datasets and suggestions from local stakeholders. Stakeholder surveys also played a key role in providing strategic direction to this process. For example, characteristics of cases and those being tested, such as age, sex, ethnicity, deprivation index, and presence of disease symptoms, were included in the LA Report following requests in an end-user survey in 2020. Updates to the reports were communicated to stakeholders in the surveillance outputs and through the web platform where reports were shared. The evaluation described here was part of this process of review and development. We aimed to explore how stakeholders in LAs used the OST surveillance reports and data outputs, the extent to which resources influenced local decision-making, and to identify potential areas of development that stakeholders would like to see included in future reports.

## Methods

A questionnaire was developed that explored five themes: (i) how the OST reports were used; (ii) how the surveillance outputs had influenced local intervention strategies; (iii) the timeliness of updates to surveillance reports; (iv) whether information on significant aspects of the epidemic had been included in the reports and additional data needs; and (v) future developments that stakeholders would like to be included in the reports. Closed and open questions were used to capture information concerned with practice, advice, context, and detail around operational use. For those questions that explored metrics, examples of the figures included in the report were provided (supplementary material, Appendix 1).

The questionnaire (supplementary material, Appendix 1) was created using a Public Health England web-based questionnaire tool (SelectSurvey). Before implementation, the draft questionnaire was piloted with four people that had participated in a previous evaluation in late 2020. The questionnaire was revised in line with the comments received from this pilot study.

On 3 November 2021, the dissemination list for the UKHSA COVID-19 Situational Awareness Explorer Portal, which has the same target audience as the OST surveillance reports, was used to distribute invitations to take part in the survey. The 2,400 people who were invited to take part were employed by LAs in a variety of roles, including directors of public health, consultants in public health, public health analysts, information analysts and epidemiologists. Three hundred out-of-office notifications were received, and 75 emails were undeliverable. A reminder was sent two weeks later, and the survey closed after a total of four weeks. Respondents could be anonymous or provide details for further contact. Questionnaire responses were anonymised and data aggregated. The data were processed and analysed using R [2].

### Patient & public involvement

No patients were involved in this study.

## Results

### Questionnaire responses

In total, 366 people participated in the survey, corresponding to a response rate of 15.3%. Only 66 people completed the entire questionnaire. Consequently, the number of answers varied between questions. The number of responders is indicated in the results and titles of the figures and tables. Most participants worked in public health, data science, epidemiology, or business intelligence. Positions within respondents’ organisations (number of respondents (n) = 129) included analysts and officers within their specialist fields (36.4%), managers (13.2%), consultants in public health (10.9%), lead and head roles (11.6%), and assistant directors and directors of public health (5.2%). All nine UKHSA regions were represented among the respondents (supplementary material, Figure S1). Out of those who indicated work place (n = 142), 44.4% worked at county councils (Upper Tier Local Authorities), 31.0% at unitary authorities, 19.0% at district councils (Lower Tier Local Authorities), and 1.4% with Health Protection Teams or Public Health England (PHE) centres (supplementary material, Figure S1).

### How were the UKHSA OST outputs used?

**Fig. 1:**
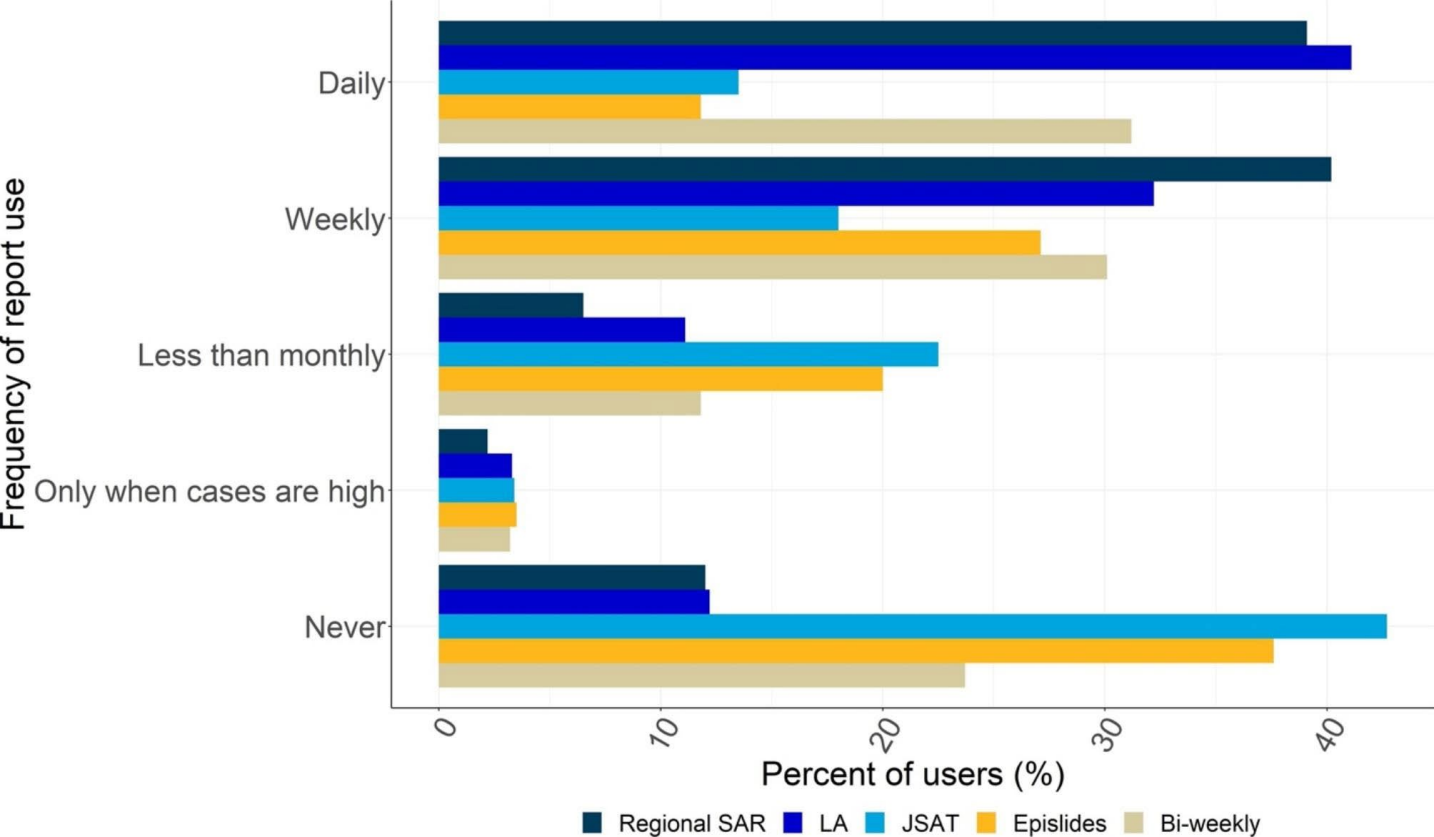
Frequency with which Local Authorities used five UKHSA Outbreak Surveillance Team reports (response n = 93). For description of the reports, please see supplementary material, Table S1

**Fig. 2:**
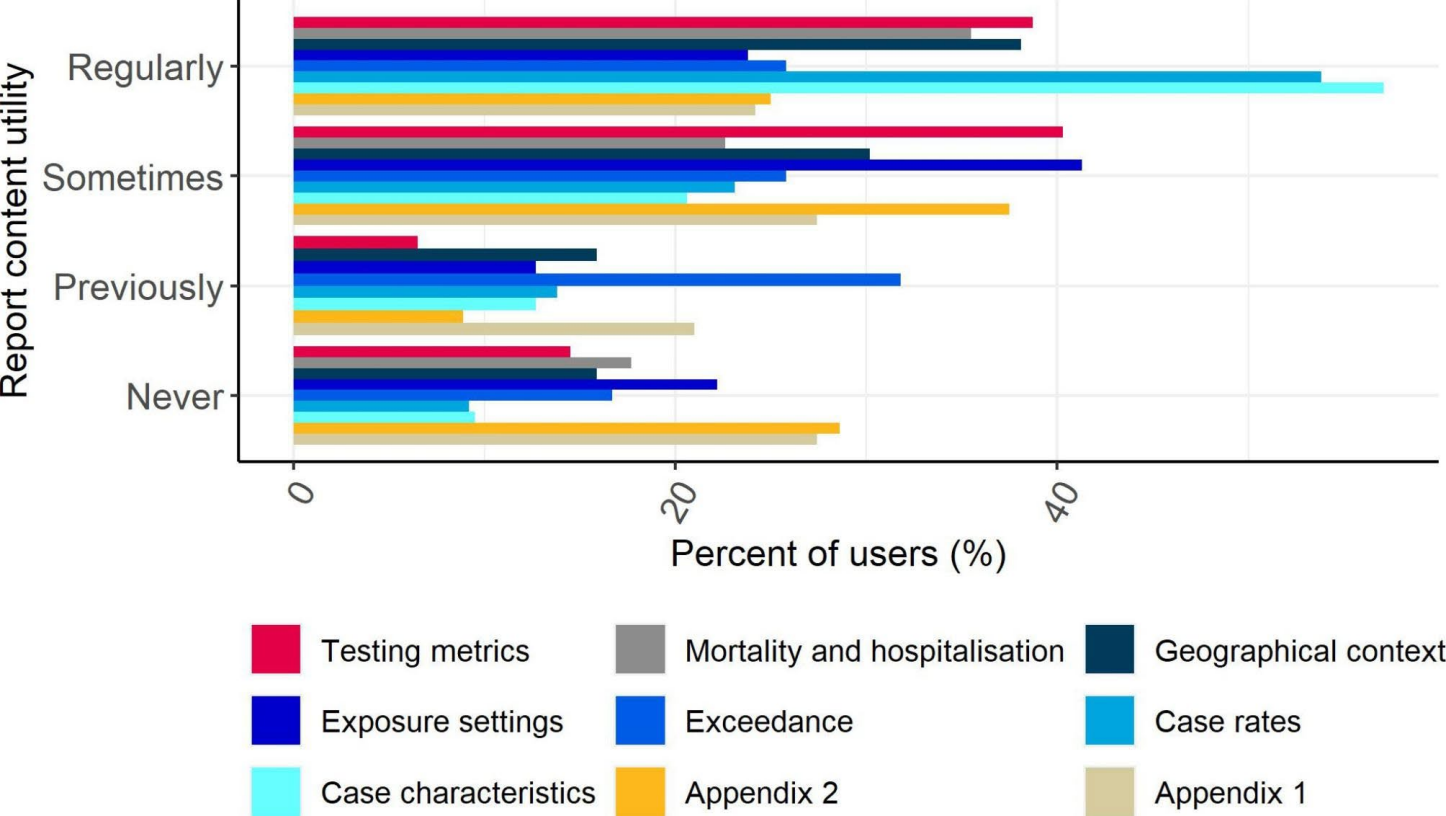
Frequency with which Local Authorities used each of the sections of the LA Report (n = 66). For description of the LA report, please see supplementary material, Table S2

**Fig. 3:**
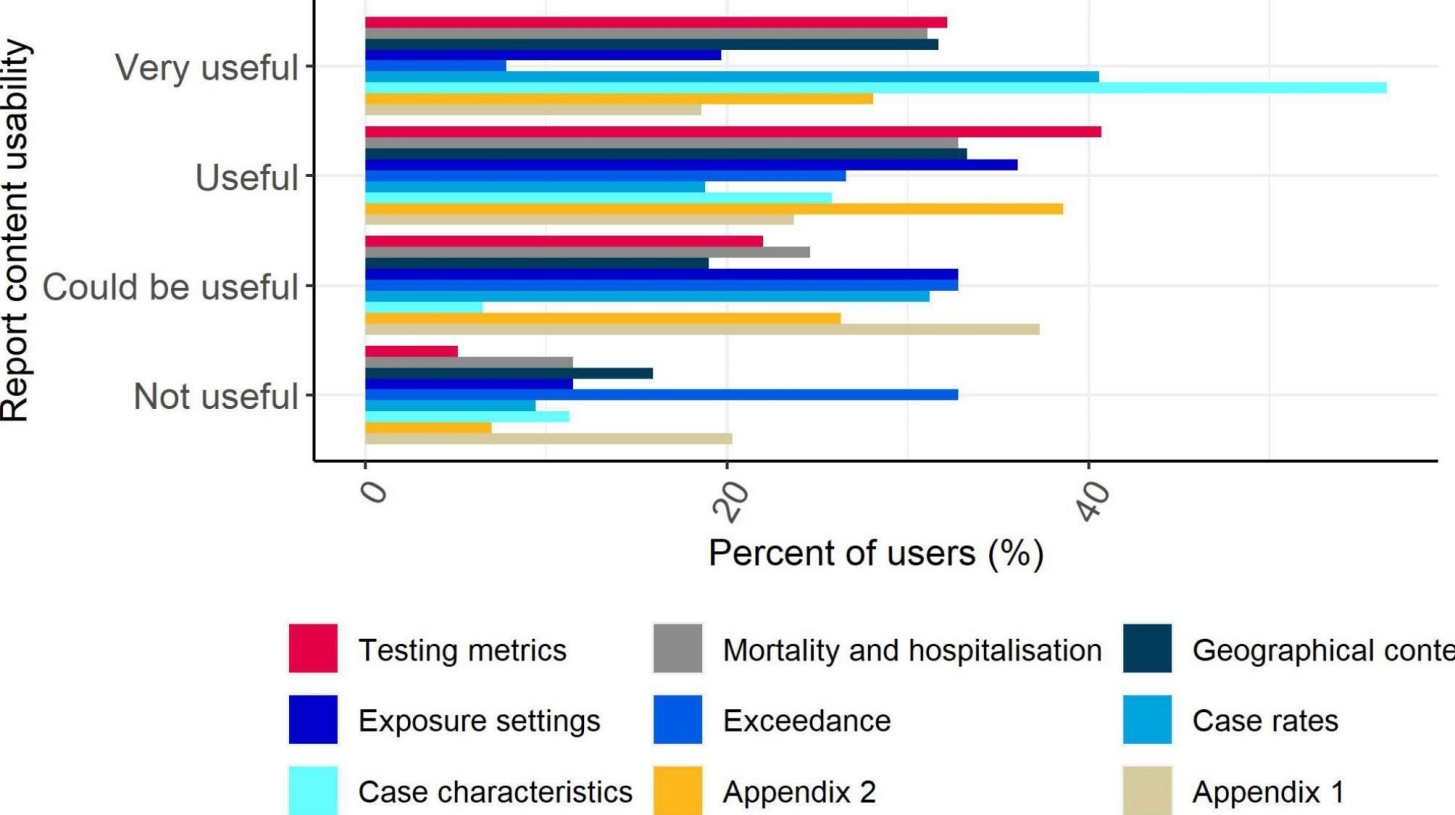
How useful were the resources included within the LA Report? (n = 66). For description of the LA report, please see supplementary material, Table S2

The most commonly used OST outputs were the LA Report and Regional Situational Awareness Report (SAR). Over 70% of contributors used them either on a daily or weekly basis (Fig. 1). Within the LA Reports, the most frequently used sections were case characteristics, case rates and testing metrics (Fig. 2). Over 40% of responders considered the case characteristics and case rates as ‘*very useful’*, as did over 30% of those who responded to the questions concerned with testing metrics, geographical context and mortality and hospitalisations (Fig. 3). The Joint Situational Awareness Team (JSAT) and Epislide reports were used less frequently (18% weekly and 27% weekly, respectively).

The frequency with which the reports were consulted was not related to the local burden of SARS-CoV-2 cases: less than 5% of respondents only used the surveyed reports when cases where high (Fig. 1). The reports provided LAs with additional context for local COVID-19 epidemiology and were a source for charts and figures which LAs added to local reports and presentations (Table 1). For example, more than 50% of the participants consulted the Regional SAR Report, LA Report and Daily Regional Reports for daily information concerned with local developments in the epidemic (Table 1). This information was also used to develop control and intervention strategies, such as the introduction of diagnostic services and the use of face masks in school. However, some responders (2.1%) indicated that they had started to utilise data from the UKHSA COVID-19 Situational Awareness Explorer Portal in preference of the OST reports.

**Table 1:** Use of UKHSA Outbreak Surveillance Team reports by Local Authorities (number of respondents n = 94)

	Daily regional reports				Regional SAR				LA report				JSAT report				Epislide report		
	%		n		%		n		%		n		%		n		%		n
Main source of daily updates on local COVID-19 epidemiology	23.4		22		33.0		31		25.5		24		8.5		8		5.3		5
Source of additional context for local COVID-19 epidemiology	54.3		51		57.4		54		59.6		56		34.0		32		42.6		40
Source of charts/figures for local reports/presentations	30.9		29		37.2		35		47.9		45		16.0		15		19.1		18
Summarise key information to forward to partners	21.3		20		31.9		30		31.9		30		9.6		9		12.8		12
Forwarded in entirety to partners	4.3		4		4.3		4		5.3		5		3.2		3		7.4		7
I do not use the report	27.7		26		16.0		15		14.9		14		50.0		47		50.0		47
Other	2.1		2		3.2		3		2.1		2		3.2		3		3.2		3

### How did the local SARS-CoV-2 reports influence intervention strategies?

**Fig. 4:**
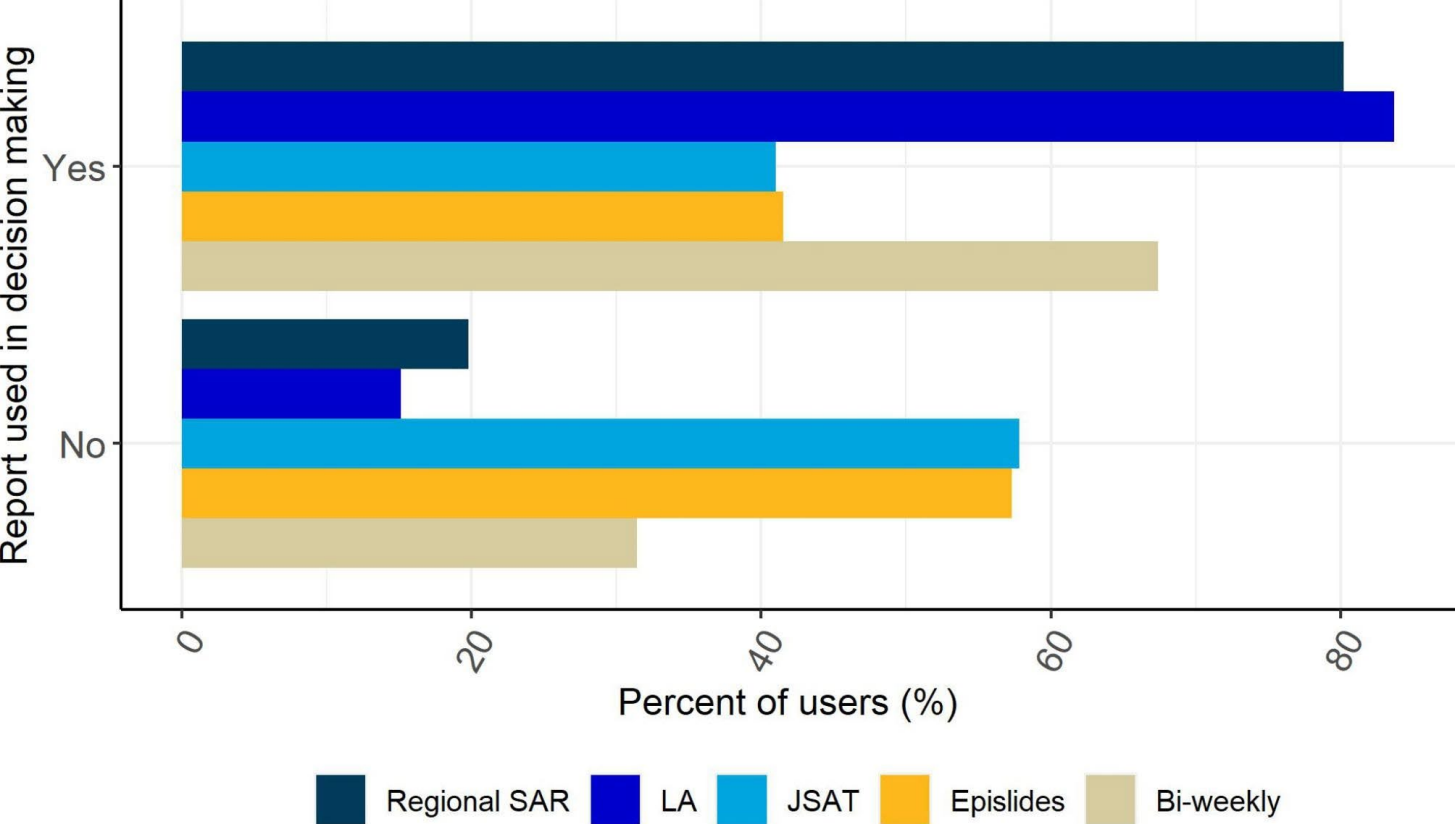
Proportion of contributors who used information provided in the reports to inform decision making within their organisation (n = 66). For description of the reports, please see supplementary material, Table S1

Most people had used the LA Report (84%), Regional SAR (80%) and Bi-weekly report (67%) for decision making within their organisations (Fig. 4). More than 40% had used the JSAT and Epislide reports for this purpose. The surveillance resources were also used as an evidence base to inform intervention strategies and to answer enquiries. Out of 63 responders, 68% had instigated changes to COVID-19 intervention strategies as a result of the information presented in the reports. The reports had influenced the timing of interventions; targeted communications and diagnostic testing; supporting specific areas and populations by providing mobile testing sites; and guiding decisions concerning the provision of testing in schools and use of non-pharmaceutical interventions, such as face masks.

### Timeliness of information included in reports

Most respondents considered that the surveillance content was flexible and had responded to the evolving demands of the epidemic response in a timely manner. However, the timeliness of content updates and, consequently, the ability to obtain up-to-date insight into pandemic developments varied between reports: 56% (n = 66) considered that revisions to the JSAT reports were made in a timely manner compared to 81% for the regional SARs. Some stakeholders were concerned that the most recent population census estimates used to calculate case rates by ethnicity were outdated. Consequently, recent changes in the profile of local populations had not been reflected in the estimates that had been released. It was also felt that the provision of vaccination data had been delayed and that the reduction in reporting frequency over time had impacted local reporting.

### Additional data needs not covered in the reports

Of 57 responders, 44% suggested that additional information would be useful to support decision making in the autumn of 2021. Of those who requested additional indicators or information (n = 25), vaccination and hospitalisation data were requested by 44% and 24%, respectively. It was also suggested that information on the date of testing, repeat infections, ethnicity, infection during pregnancy, underlying health conditions, school absence and information from wastewater testing could be included.

### Future developments

OST surveillance reports were produced in an automated format as often as daily to once a week. However, as the demand for information evolved, some local organisations developed their own in-house epidemiological intelligence reports alongside the OST resources. The COVID-19 Situational Awareness Explorer Portal, which was introduced in December 2020, provided access to interactive data and underlying databases, allowing greater flexibility in the ways that information could be processed and disseminated to local decision makers. The COVID-19 Situational Awareness Explorer Portal was used by 70% of responders. The dashboard and local reporting systems were used by some participants as their primary sources of information. Of 56 respondents, 89% said that the information requirements of their organisation would be met if the LA Reports were incorporated into the dashboard. However, 10.7% (n = 6), expressed concerns about this strategy and cited issues they had encountered with limited access to the dashboard, the time taken to navigate the interface and locate information, and the limited range of file format options available when saving information. Incidents of late updates to daily data, which caused delays in the response to new cases, were also mentioned as a challenge among the users.

## Discussion

The findings of the study highlight the value of producing timely, high-quality surveillance data as a basis for public health action in an epidemic, and the role of evaluation in the development of surveillance resources. Based on the survey results, the reports produced by the UKHSA OST continued to be influential through 2021 when the Delta SARS-CoV-2 variant was dominating the UK epidemic [4]. The majority of respondents used more than one OST surveillance output on a daily or weekly basis and over 80% used them to support decision making within their LAs (Figs. 1 and 4). The content of the reports was reviewed and updated regularly to reflect changes in data availability, insights made by the OST, and stakeholder requests. As a result of the 2021 evaluation, additional epidemiological indicators requested to support the pandemic response were added to the reports together with updated data flow pathways to improve publication timelines.

Much of the information from the current evaluation was used to revise the reports in terms of content and the underlying production processes. For example, one change to the surveillance outputs was the inclusion of vaccination data. Initially, local authorities could access vaccination data on the COVID-19 Situational Awareness Explorer Portal and, from May 2022, vaccination data were included in the LA Reports. The data were updated for every LA Report produced, which gave local authorities a timely source of information with which to assess vaccination trends.

The OST surveillance outputs were produced by combining data from multiple databases managed by the UKHSA, Office for National Statistics and National Health Services. Creating the datasets to be used in reports from these resources was a complex data engineering challenge which was under constant development. Review processes allowed new sources of information to be brought into the reports and ensured that production times were optimised. For example, since this evaluation was carried out, data on SARS-CoV-2 reinfections have been introduced, which was requested by users of the OST reports. The change from only reporting one infection event in individuals to reporting episodes of infection, where a positive test was considered a new episode if at least 90 days had passed since previous infection, was introduced in January 2022. The move to episode reporting instead of case reporting reduced the size of the datasets being processed and was fully automated, thereby making production timescales more predictable. Furthermore, an online information repository was developed which allowed reports and data to be shared more easily. However, although the processing time of infection data improved, the frequency of reports has fallen due to the increased size of the testing data (USD managed by lab informatics, UKHSA).

To inform stakeholders of publication delays of reports as a result of dataflow challenges, OST posted updates on the web platform where surveillance reports are shared. This process could be improved by optimising communication within the organisation to ensure that delays affecting publication timelines of reports are communicated immediately. Improving communication with users and increasing awareness of available information is an ongoing process. For example, an OST communication channel could be established to provide an easily accessible overview of report production, offered resources, and planned developments. In addition, since some data developments suggested by stakeholders in the evaluation were already available, such as hospitalisation data, a guide consisting of an outline of available reports and the COVID-19 Situational Awareness Explorer Portal would also facilitate the navigation of resources.

Going forward, the majority of respondents felt the data requirements of their organisations could be fulfilled by incorporating the LA report in the COVID-19 Situational Awareness Explorer Portal. Nevertheless, concerns were raised about navigating information on the dashboard and summarising data in an accessible format. Additional discussions with both users and non-users of the dashboard would be required to identify and address potential barriers.

### Strengths and weaknesses of the study

The small sample size was the main weakness of the study, with only 15.3% of the COVID-19 Situational Awareness Explorer Portal dissemination list taking part in the study. Furthermore, the use of the dashboard email list for distribution of the questionnaire may have resulted in overrepresentation of dashboard users among study participants. Nevertheless, all UKHSA regions were represented among the respondents and the responses were consistent in terms of detailed insight into the opinions of users which were then used to develop and maintain the surveillance systems to support LAs in the SARS-CoV-2 epidemic response. In addition to regular quantitative evaluations, qualitative interviews with local partners could be carried out for a more in-depth review of stakeholders’ priorities.

## Conclusions

The establishment of a dedicated outbreak surveillance team to provide epidemiological intelligence to English LAs contributed to the effectiveness of the SARS-CoV-2 pandemic response. This evaluation showed that the local surveillance reports were a valuable and impactful resource used by local stakeholders as they responded to the epidemic. Regular evaluation maintained and improved the quality of the UKHSA OST working practices. Current policy that affects disease epidemiology and monitoring requirements, such as vaccine uptake, needs to be considered in the continuous development of surveillance reports. Based on feedback provided by stakeholders, we revised the epidemiological indicators reported and optimised data flow pathways. This ensured that the OSTs surveillance outputs remained relevant to the development of control and intervention strategies by stakeholders throughout the pandemic. The methods developed for the production of the SARS-CoV-2 reports have already been applied as part of the subsequent national response to the mpox outbreak [5] and investigation of severe acute hepatitis of unknown aetiology in children [6]. The lessons learnt will be a valuable resource for the UKHSA and others as they plan future surveillance systems for endemic and epidemic infections.

## Electronic supplementary material

Below is the link to the electronic supplementary material.

Supplementary Material 1

Supplementary Material 2

## Data Availability

The anonymous dataset collected and analysed during the current study is available from the corresponding author on reasonable request.

## List of abbreviations

DPH: Directors of Public Health
IMT: incident management teams
JSAT: Joint Situational Awareness Team
LA: Local Authority
NHS: National Health Service
ONS: Office for National Statistics
OST: Outbreak Surveillance Team
PHE: Public Health England
SAR: Situational Awareness Report
SARI: Severe Acute Respiratory Infection
SARS-CoV-2: Severe acute respiratory syndrome coronavirus 2
SGSS: Second Generation Surveillance System
UK: United Kingdom
UKHSA: UK Health Security Agency
USD: Unified Sample Database
