# Supplementary material for: Influence of SARS-CoV-2 surveillance outputs produced by the UK health security agency (UKHSA) outbreak surveillance team on decision-making by local stakeholders
Source: BMC Public Health. 2023 May 22;23:926. doi: 10.1186/s12889-023-15784-8 (PMC10202050; doi:10.1186/s12889-023-15784-8)
Supplement: Supplementary file 1 — Supplementary Material 1 [file 12889_2023_15784_MOESM1_ESM.pdf]

Supplementary material for

Influence of SARS-CoV-2 surveillance outputs produced by the UK Health Security Agency (UKHSA) Outbreak Surveillance Team on decision-making by local stakeholders

Katriina Willgert[1,2,] Jo Hardstaff[1], Stephanie Shadwell[3], Alex Bhattacharya[1], Paula Blomquist[1], Roberto Vivancos[1], Ian Simms[1]

[1]*Outbreak Surveillance Team, Field Services, UK Health Security Agency, United Kingdom*
[2]*Disease Dynamics Unit, Department of Veterinary Medicine, University of Cambridge, United Kingdom*
[3]*Data Product Development, Data Operations, UK Health Security Agency, United Kingdom*

Supplementary tables

Table S1. Description of SARS-CoV-2 surveillance reports produced by the UK Health Security Agency Outbreak Surveillance Team in November 2021 to support local stakeholders.

Report	Background	Geographical resolution	Frequency of production	Report summary	Format	Data sources
Bi-weekly report	Regional report for LAs.	National, UKHSA region*, NHS region, UTLA, MSOA, LA	Twice weekly	Daily cases, cumulative total, 7-day moving average, epidemic curves, epidemic curves by pillar, individuals tested by pillar, 7-day test positivity, test positivity by pillar, test positivity by pillar and age group, 7-day case rate, 7-day case rate funnel plot, cumulative rate, case rate map, 5% of MSOAs with the highest case rates for the last one and two weeks, Test and Trace exposure setting, outbreaks setting, ethnicity of cases, age and sex distribution of cumulative cases (pillar 2 only), deaths by setting, ONS COVID-19 deaths by setting, COVID-19 deaths in hospitals, NHS England-reported percentage change in deaths and deaths by age group. Syndromic data: GP, NHS111 and Emergency department syndromic surveillance for acute respiratory infection and COVID-19 like illness, Bing search engine searches for "cough" and "fever" compared to expected levels. Data used to compile the report were also provided.	Word report and accompanying data	SGSS, HPZone, NHS England COVID-19 daily deaths, NHS 111 Syndromic Surveillance, EDSSS, ONS England COVID-19 weekly deaths summary, GP Out-of-Hours Syndromic Surveillance, ONS population data
Epislides	First produced in September 2020 for the Joint Biosecurity Centre (JBC) for bronze (operational), silver (tactical) and gold (strategic) meetings held, but were then also produced for local authorities and field services.	UKHSA region*, JBC subregional levels, LA	Weekly	Cases, daily cases by pillar, 7-day moving average, epidemic curves, individuals tested, test rate, test positivity, case rate, case rate heat map, case rate map, case rate by age group, 7-day moving average in 60+ group, case rate heat maps by age group, case age-sex pyramids. Accompanying data were originally provided.	PowerPoint presentation at UKHSA regional level and separate PowerPoint presentations for each JBC subregion.	SGSS, USD, ONS population data, HPZone

Report	Background	Geographical resolution	Frequency of production	Report summary	Format	Data sources
JSAT Joint situational awareness report	The initial national situational awareness report was requested by the COVID-19 IMT and Incident Director in June 2020. Intended audience at the time was IMT, Incident Director, Department of Health, Cabinet Office, the Scientific Advisory Group for Emergencies (SAGE) and PHE Regional Directors.	National, UKHSA region*, LTLA, LA	Daily except Sundays	Cases by test, daily cases, weekly cases, symptomatic cases, variant case numbers, test rate, test positivity, case rate, change in case rates, case rate map, LTLAs with the highest case rates, common exposures, ethnicity of cases, test positivity for age groups 11-16 years, 17-21 years and 60+, case rate for age groups 11-16 years, 17-21 years and 60+, incidents of acute respiratory infection, hospitalisation, mortality, deaths by place. Vaccine uptake at a national and UKHSA regional level.	PowerPoint presentation and accompanying data.	SGSS, NHS England & Improvement COVID-19 Hospital Activity Data, COVID-19 SARI-Watch,
Local authority (LA) report	Report provided to LAs for the purpose of monitoring and supporting the management of COVID-19 infections in LAs. Initially only produced for LAs with a red exceedance rating of expected SARS-CoV-2 incidence. By the end of September 2020, they were produced for all Local Authorities	National, LTLA, LA, LSOA, wards	Mondays, Tuesdays, and Thursdays	Testing metrics, case rate, exceedance trajectories, geographical context, contact tracing data, exposure setting, outbreaks setting, case characteristics, hospitalisation, mortality. For additional information on content, please see Supplementary material, Table S2.	Semi-automated Word report produced in R (R Core Team, 2021) and automatically uploaded to web-based platform shared with LAs.	HPZone, UKHSA EpiCell linelist, SGSS, USD. NHS hospitalisations, ONS mortality and populations
Regional situational awareness report (SAR)	Requested by the director of the National Infection Service, following requests from regional teams in July 2020. Target audience were regional PHE teams and DsPH.	LTLA, LA	Daily except Sundays	Test rate, test positivity, weekly test positivity, case rate, weekly case rate, 7-day change in case rates. Maps of weekly test positivity, 7-day moving average testing rate, weekly case rate, and 7-day change in case rate. Exceedance trajectories, red-amber-green (RAG) rating, weekly community outbreaks.	Word document with accompanying data	SGSS, HPZone, UKHSA COVID-19 daily linelist, USD, ONS population data, NHS hospitalisations

*previously referred to as PHE centres (East Midlands, East of England, London, North East, North West, South East, South West, West Midlands, Yorkshire and Humber)
PHE = Public Health England, IMT = Incident Management Team, DsPH = Directors of public health,
LSOA = Lower Layer Super Output Areas, LTLA= lower-tier local authorities, MSOA = Middle Layer Super Output Area, UTLA = upper-tier local authority, LA = Local Authority,
GP = general practitioner, NHS 111 = Phone service for medical advice or online assessment,
ONS = Office for National Statistics, SGSS = Second Generation Surveillance System, EDSSS = Emergency Department System Syndromic Surveillance, USD = Unified Sample Database

Table S2. Description of the different sections in the Local Authority (LA) report, November 2021.

Section	Description
Case characteristics	Age and sex pyramids of confirmed cases for the most recent reporting period and over a longer time period, heatmaps of case rates by age, ethnicity, IMD, test positivity by age and proportion of cases with symptoms.
Case rates	Epidemic curves of cases, case rates, positivity and testing rates by pillar*. A table of current case numbers and positivity and recent change, as well as an overview of number of cases, number of tests, case rates, testing rates and PCR positivity in the LA, other neighbouring LAs, the region as a whole and the entire country by pillar* in the most recent reporting period and over a longer time period.
Exceedance	Exceedance time-series illustrating daily case rate in the LA over the most recent 14 days with expected trajectory of case rates based on the previous 6 weeks.
Exposure settings	Exposure indicated by residence type and activity setting. Based on contact tracing data of settings visited by confirmed cases 3 to 7 days prior to onset of symptoms.
Geographical context	Case rates for most recent and prior weeks for below and above 60 year olds, maps of case numbers by Lower Layer Super Output Area (LSOA) and by ward.
Mortality and Hospitalisation	Mortality by setting (care home, home, hospital, hospice, other communal establishment, or elsewhere), 60-day mortality rates and hospitalisation numbers.
Testing metrics	Epidemic curves of individuals tested by pillar, testing rates by age, ethnicity and IMD.
Appendix 1	Details of outbreaks, clusters, exposures and issues in the LA, including information about the location, setting type, number of cases and persons exposed. Cases in private residencies are excluded.
Appendix 2	Testing rates for different age groups by pillar*.

*Pillar 1: tests processed in UKHSA laboratories or NHS hospitals, which mainly includes tests of symptomatic individuals seeking care. Pillar 2: tests from community-based testing
LA= Local Authority
IMD= index of multiple deprivation

Supplementary figures

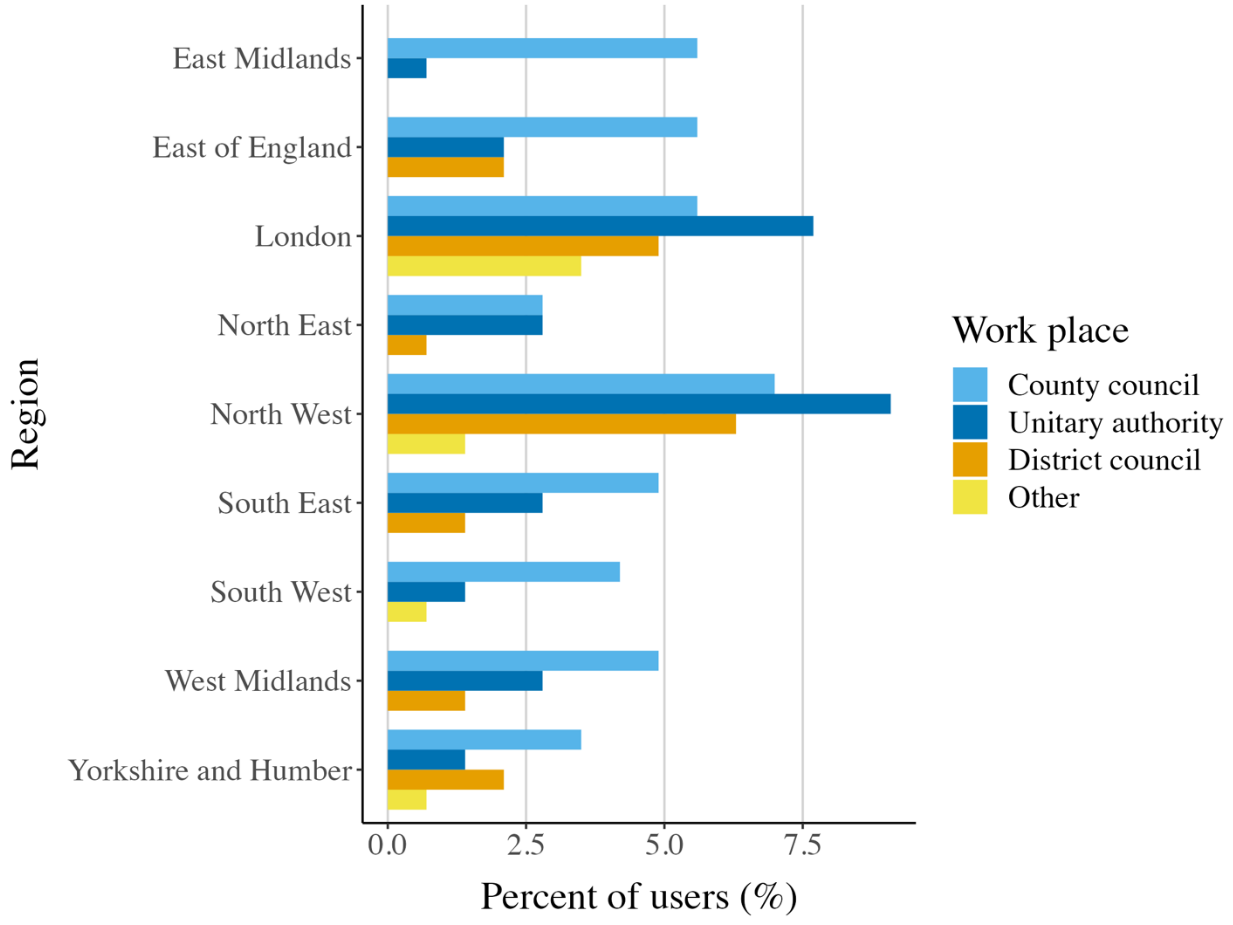

Figure S1. UKHSA region (previously referred to as PHE centres) and work place of the survey respondents (n=143).
