# Supplementary material for: Influence of SARS-CoV-2 surveillance outputs produced by the UK health security agency (UKHSA) outbreak surveillance team on decision-making by local stakeholders
Source: BMC Public Health. 2023 May 22;23:926. doi: 10.1186/s12889-023-15784-8 (PMC10202050; doi:10.1186/s12889-023-15784-8)
Supplement: Supplementary file 2 — Supplementary Material 2 [file 12889_2023_15784_MOESM2_ESM.pdf]

Supplementary material for

Influence of SARS-CoV-2 surveillance outputs produced by the UK Health Security Agency (UKHSA) Outbreak Surveillance Team on decision-making by local stakeholders

Katriina Willgert[1,2,] Jo Hardstaff[1], Stephanie Shadwell[3], Alex Bhattacharya[1], Paula Blomquist[1], Roberto Vivancos[1], Ian Simms[1]

[1]*Outbreak Surveillance Team, Field Services, UK Health Security Agency, United Kingdom*
[2]*Disease Dynamics Unit, Department of Veterinary Medicine, University of Cambridge, United Kingdom*
[3]*Data Product Development, Data Operations, UK Health Security Agency, United Kingdom*

Appendix 1. Questionnaire

Evaluation of the influence of the OST SAR-CoV-2 surveillance outputs on the development of intervention strategies used by Local Authorities

Introduction

Evaluation the influence of the UK-HSA Outbreak Surveillance Team SAR-Cov-2 surveillance outputs

Dear colleagues,

We would like to invite you to complete a questionnaire to help us evaluate the impact of COVID-19 surveillance outputs produced by the UK-HSA Outbreak Surveillance team (OST) on intervention strategies and plan for the future.

The format of our surveillance outputs will be updated to meet evolving needs of stakeholders and assure appropriate allocation of resources. This evaluation is an opportunity for stakeholders to have their say on the format of future surveillance outputs. The questionnaire should take ten minutes to complete. All data will be stored securely and the results of the survey will be anonymous.

Please contact [email address] if you have any questions.

Thank you in advance for completing the questionnaire, we look forward to hearing your thoughts.

Best wishes,

Outbreak Surveillance Team
UK-HSA

COVID-19 response

1. Have you taken part in the COVID-19 pandemic response?*

- ○ Yes
- ○ No

If answered "No" to question 1:

Thank you

Please forward the questionnaire to appropriate colleague in your organisation involved in the COVID-19 pandemic response.

Thank you for your help and collaboration.

If answered "yes" to question 1:

About you

2. What is your current position?

- ○ Deputy regional director
- ○ Director of Public Health
- ○ Regional director
- ○ Other, please specify

3. What is your specialty?
Select at least 1.

- ☐ Data science
- ☐ Epidemiology
- ☐ Microbiology
- ☐ Public health
- ☐ Other, please specify

4. Where are you based?

- County council (upper tier local authority)
- Unitary authority
- District council (lower tier local authority)
- Health Protection Team/PHE centre
- Other, please specify

5. Which Public Health England region does your workplace belong to?

- East Midlands
- East of England
- London
- North East
- North West
- South East
- South West
- West Midlands
- Yorkshire and Humber

Report use

6. How often do you use or refer to each of the following reports?

	**Daily**	**Weekly**	**Less than monthly**	**Only when cases are low**	**Only when cases are high**	**Never**
Daily regional surveillance						
Regional situation awareness reports						
LA Reports						
JSAT						
Epislides						

7. What do you use the UK-HSA COVID-19 Daily regional surveillance report for? Please select all that apply
Select at least 1.

- ☐ Main source of daily updates on local COVID-19 epidemiology
- ☐ Source of additional context for local COVID-19 epidemiology
- ☐ Source of charts/figures for local reports/presentations
- ☐ Forwarded in entirety to partners
- ☐ Summarise key information to forward to partners
- ☐ I do not use the report
- ☐ Other, please specify

8. What do you use the UK-HSA COVID-19 Regional situation awareness reports for? Please select all that apply
Select at least 1.

- ☐ Main source of daily updates on local COVID-19 epidemiology
- ☐ Source of additional context for local COVID-19 epidemiology
- ☐ Source of charts/figures for local reports/presentations
- ☐ Forwarded in entirety to partners
- ☐ Summarise key information to forward to partners
- ☐ I do not use the report
- ☐ Other, please specify

9. What do you use the UK-HSA COVID-19 LA Reports for? Please select all that apply
Select at least 1.

- ☐ Main source of daily updates on local COVID-19 epidemiology
- ☐ Source of additional context for local COVID-19 epidemiology
- ☐ Source of charts/figures for local reports/presentations
- ☐ Forwarded in entirety to partners
- ☐ Summarise key information to forward to partners
- ☐ I do not use the report
- ☐ Other, please specify

10. What do you use the UK-HSA COVID-19 JSAT report for? Please select all that apply
Select at least 1.

- [ ] Main source of daily updates on local COVID-19 epidemiology
- [ ] Source of additional context for local COVID-19 epidemiology
- [ ] Source of charts/figures for local reports/presentations
- [ ] Forwarded in entirety to partners
- [ ] Summarise key information to forward to partners
- [ ] I do not use the report
- [ ] Other, please specify

11. What do you use the UK-HSA COVID-19 Epislides for? Please select all that apply
Select at least 1.

- [ ] Main source of daily updates on local COVID-19 epidemiology
- [ ] Source of additional context for local COVID-19 epidemiology
- [ ] Source of charts/figures for local reports/presentations
- [ ] Forwarded in entirety to partners
- [ ] Summarise key information to forward to partners
- [ ] I do not use the report
- [ ] Other, please specify

Public health impact of OST outputs

12. Has the information provided within OST outputs been used for decision making within your organisation?

	Yes	No
Daily Regional Surveillance	○	○
Regional Situational Awareness Reports	○	○
LA Reports	○	○
JSAT	○	○
Epislides	○	○

13. Have any changes in COVID-19 intervention strategies been instituted in your local region as a result of these decisions?

Yes

No

14. The content of reports has been revised during the course of the pandemic. Did these revisions maintain a timely insight into the development of the pandemic?

	Yes	No
Daily regional surveillance		
Regional situation awareness reports		
LA Reports		
JSAT		
Epislides		

15. If the content of reports did not maintain a timely insight into the development of the pandemic please provide details of where they could be improved.

UK-HSA COVID-19 LA Report: case numbers and rates

16. Utility of tables and figures in the PHE COVID-19 LA Report to your organisation:
How often do you use the resource below? Please select the option that applies

	Never	Previously	Sometimes	Regularly
Case numbers and rates				

17. How useful do you find the resource below? Please select the option that applies

	Not useful	Could be useful	Useful	Very useful
Case numbers and rates				

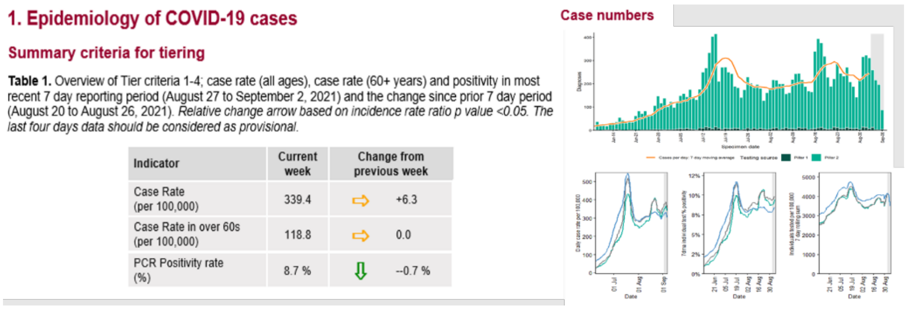

1. Epidemiology of COVID-19 cases

Summary criteria for tiering

Table 1. Overview of Tier criteria 1-4; case rate (all ages), case rate (60+ years) and positivity in most recent 7 day reporting period (August 27 to September 2, 2021) and the change since prior 7 day period (August 20 to August 26, 2021). *Relative change arrow based on incidence rate ratio p value <0.05. The last four days data should be considered as provisional.*

Indicator	Current week	Change from previous week	
Case Rate (per 100,000)	339.4	⇨	+6.3
Case Rate in over 60s (per 100,000)	118.8	⇨	0.0
PCR Positivity rate (%)	8.7 %	⇩	--0.7 %

UK-HSA COVID-19 LA Report: exceedance

18. Utility of tables and figures in the PHE COVID-19 LA Report to your organisation:
How often do you use the resource below? Please select the option that applies

	Never	Previously	Sometimes	Regularly
Exceedance	◌	◌	◌	◌

19. How useful do you find the resource below? Please select the option that applies

	Not useful	Could be useful	Useful	Very useful
Exceedance	◌	◌	◌	◌

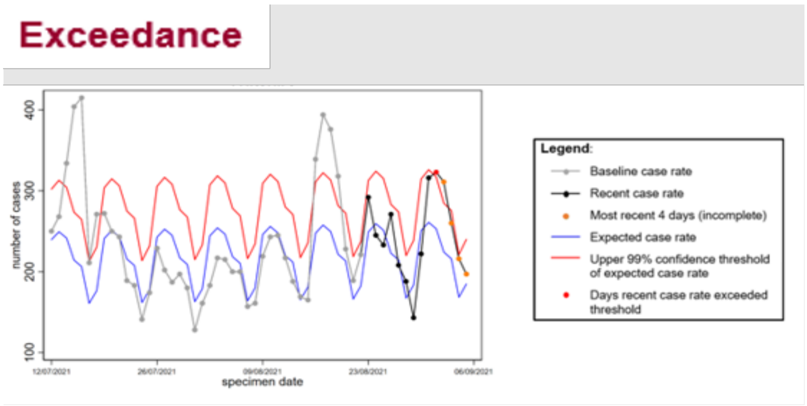

UK-HSA COVID-19 LA Report: geographical context

20. Utility of tables and figures in the PHE COVID-19 LA Report to your organisation:
How often do you use the resource below? Please select the option that applies

	Never	Previously	Sometimes	Regularly
Geographical context	◌	◌	◌	◌

21. How useful do you find the resource below? Please select the option that applies

	**Not useful**	**Could be useful**	**Useful**	**Very useful**
Geographical context	○	○	○	○

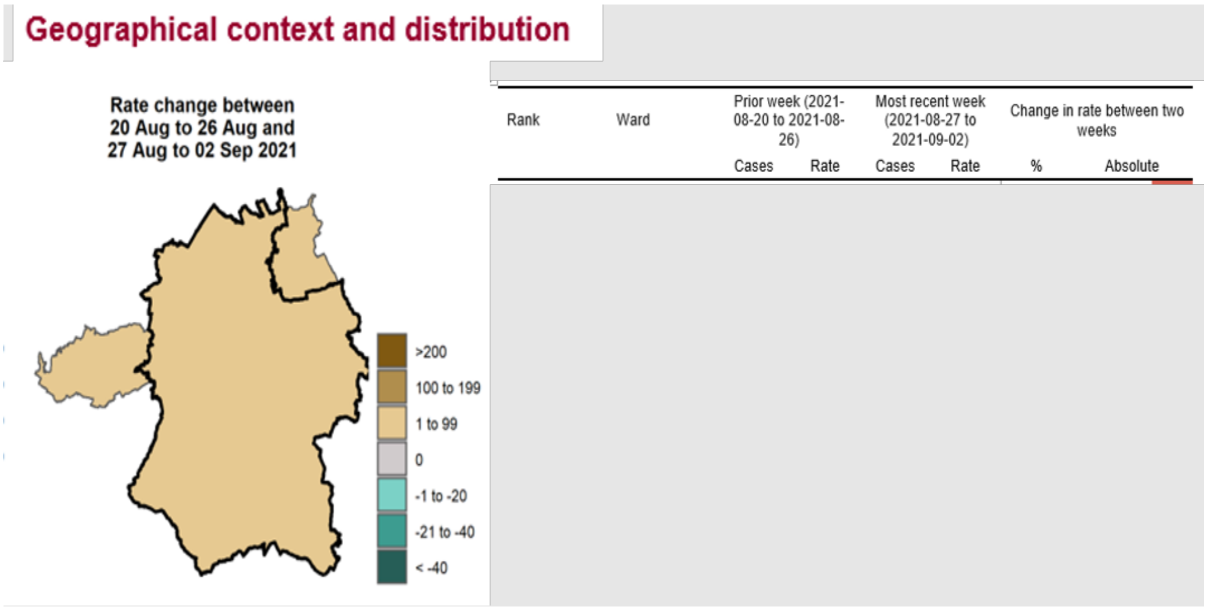

UK-HSA COVID-19 LA Report: case characteristics

22. Utility of tables and figures in the PHE COVID-19 LA Report to your organisation:
How often do you use the resource below? Please select the option that applies

	**Never**	**Previously**	**Sometimes**	**Regularly**
Case characteristics	○	○	○	○

23. How useful do you find the resource below? Please select the option that applies

	**Not useful**	**Could be useful**	**Useful**	**Very useful**
Case characteristics	○	○	○	○

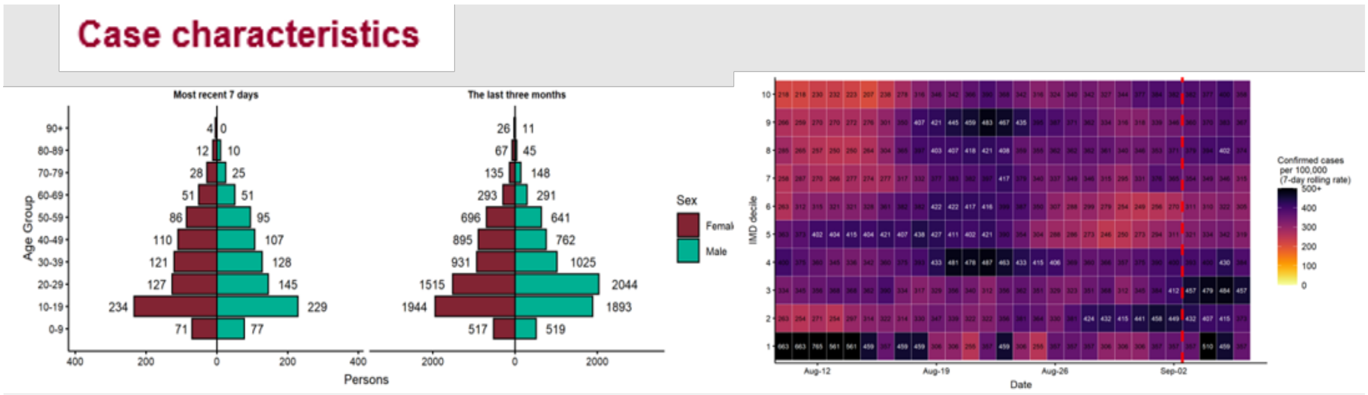

UK-HSA COVID-19 LA Report: mortality and hospitalisation

24. Utility of tables and figures in the PHE COVID-19 LA Report to your organisation: How often do you use the resource below? Please select the option that applies

	**Never**	**Rarely**	**Sometimes**	**Regularly**
Mortality and hospitalisation	◯	◯	◯	◯

25. How useful do you find the resource below? Please select the option that applies

	**Not useful**	**Could be useful**	**Useful**	**Very useful**
Mortality and hospitalisation	◯	◯	◯	◯

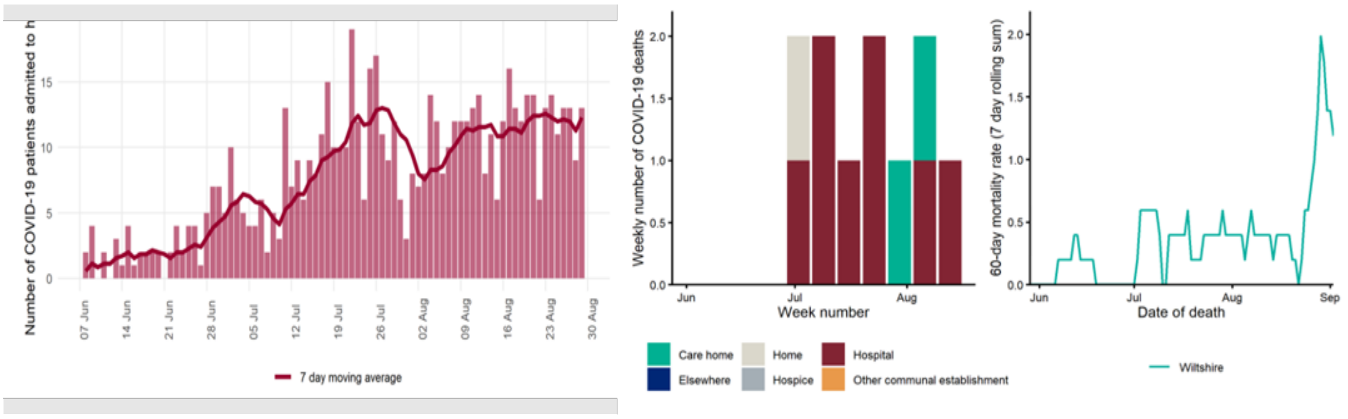

UK-HSA COVID-19 LA Report: population testing metrics

26. Utility of tables and figures in the PHE COVID-19 LA Report to your organisation: How often do you use the resource below? Please select the option that applies

	**Never**	**Previously**	**Sometimes**	**Regularly**
Population testing metrics	◯	◯	◯	◯

27. How useful do you find the resource below? Please select the option that applies

	**Not useful**	**Could be useful**	**Useful**	**Very useful**
Population testing metrics	◯	◯	◯	◯

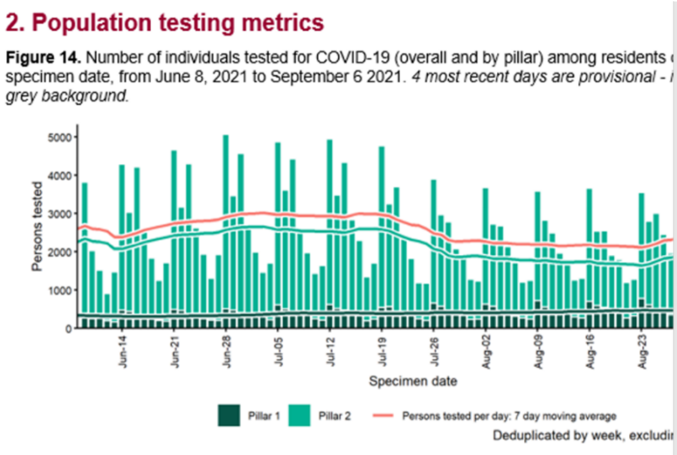

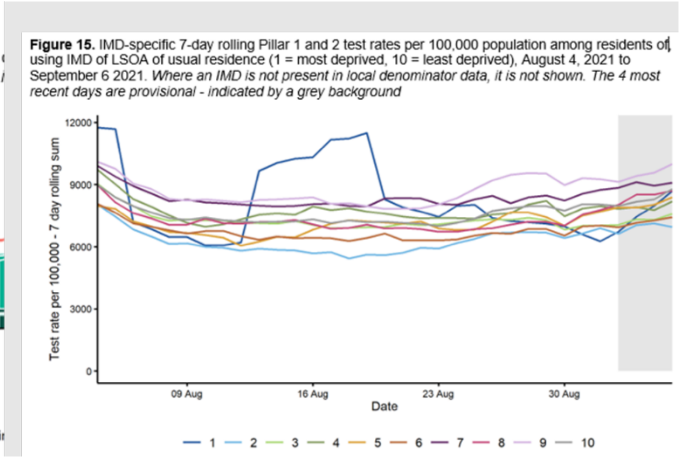

UK-HSA COVID-19 LA Report: exposure settings

28. Utility of tables and figures in the PHE COVID-19 LA Report to your organisation:
How often do you use the resource below? Please select the option that applies

	Never	Previously	Sometimes	Regularly
Exposure settings				

29. How useful do you find the resource below? Please select the option that applies

	Not useful	Could be useful	Useful	Very useful
Exposure settings				

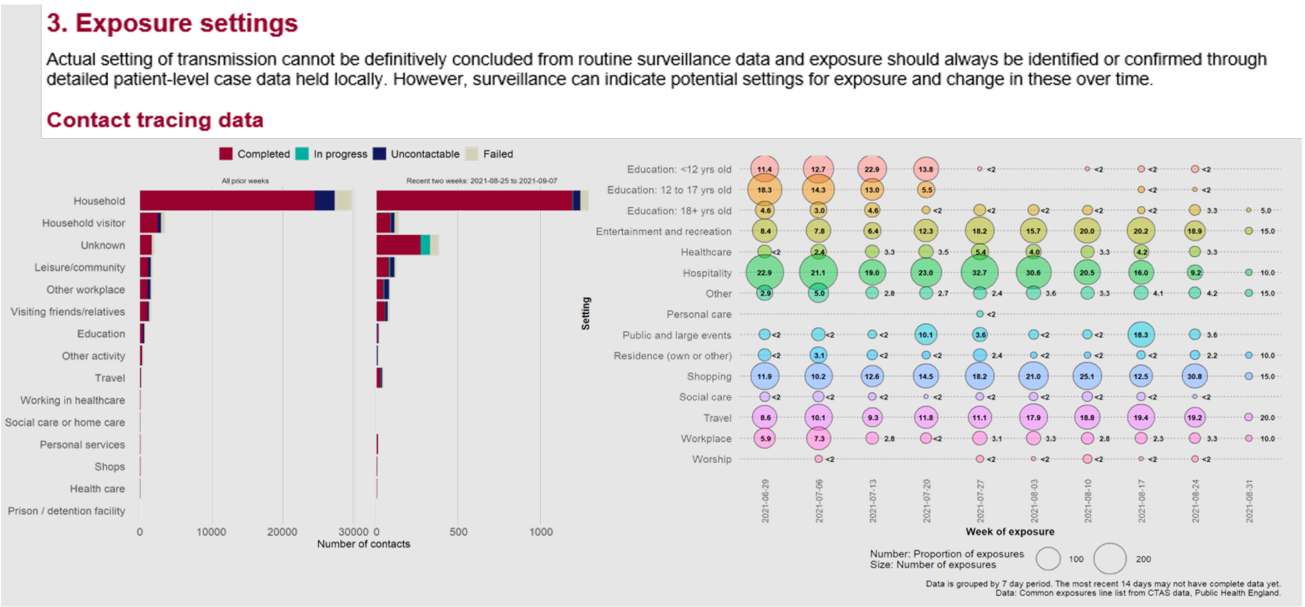

UK-HSA COVID-19 LA Report: appendices

30. Utility of tables and figures in the PHE COVID-19 LA Report to your organisation: How often do you use the resource below? Please select the option that applies

	Never	Previously	Sometimes	Regularly
Appendix 1				

31. How useful do you find the resource below? Please select the option that applies

	Never	Could be useful	Useful	Very useful
Appendix 1.				

Appendix

Appendix 1. Detail of outbreaks, clusters, exposures, issues, and threats in York with first specimen date in past 14 days or first listed in HPZone in the past 14 days (August 24 2021 to September 6 2021) - private residences excluded *Source: Residential clusters identified in linelist using case address and situations logged in HPZone. Note that HPZone information, particularly on detail related to laboratory confirmation and numbers symptomatic or exposed, usually reflects the situation when first recorded on HPZone and may not be updated.*

Location name	Postcode	Setting Type*	Situation type	Cases*	Case dates (from linelist)		HPZone information				
					Specimen date of first case	Specimen date of last case	HPZone number	Date opened	Date closed	Lab. confirmation	Persons exposed

32. Utility of tables and figures in the PHE COVID-19 LA Report to your organisation: How often do you use the resource below? Please select the option that applies

	Never	Previously	Sometimes	Regularly
Appendix 2				

33. How useful do you find the resource below? Please select the option that applies

	Not useful	Could be useful	Useful	Very useful
Appendix 2				

Appendix 2. Age-specific 7-day rolling test rates per 100,000 population by pillar among residents of
August 4, 2021 to September 6 2021. *4 most recent days are provisional - indicated by a grey backgr*

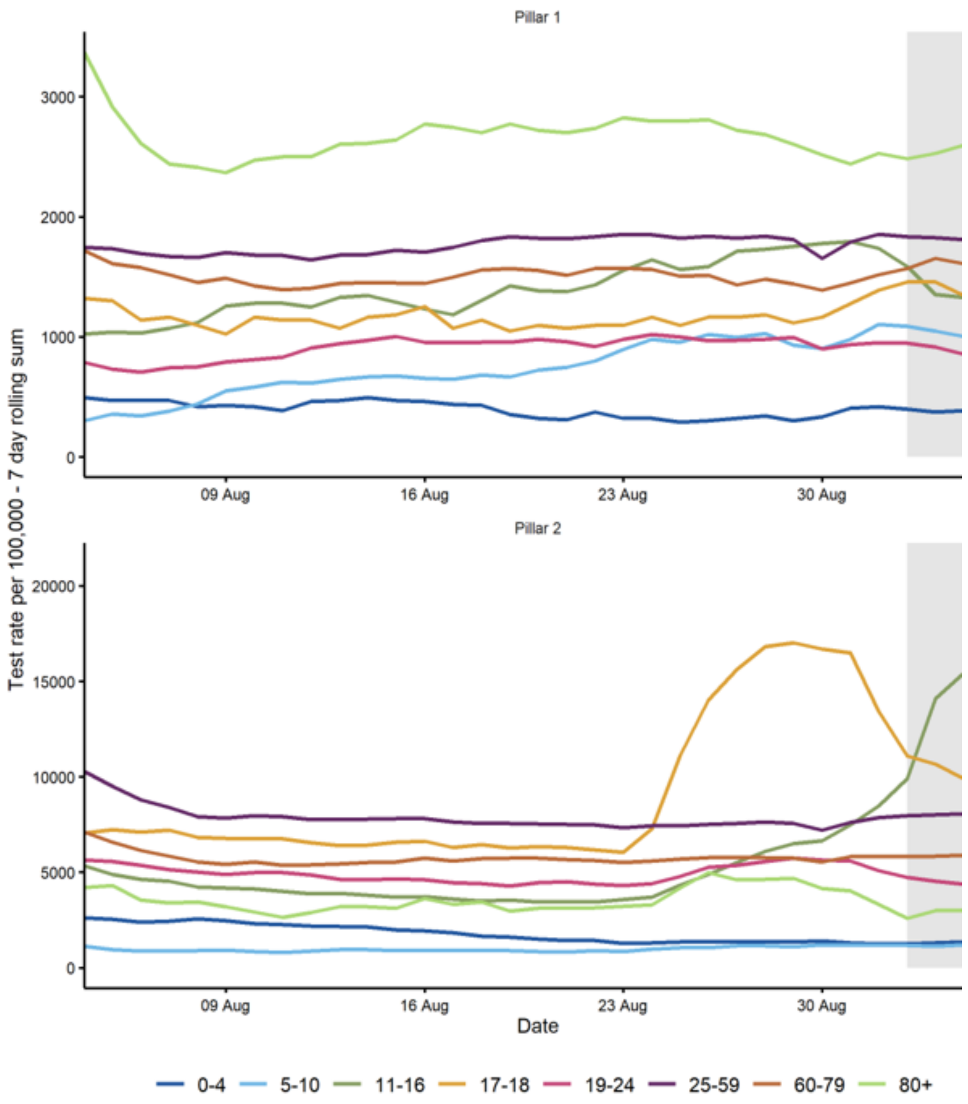

UK-HSA COVID-19 LA Report

34. Was sufficient information on populations with protected characteristics included in the reports to inform local planning?

- No
- Yes
- Please specify missing characteristics that you would have liked to have seen

35. Have you used the method companion that accompanies the LA Report?

- Yes
- No
- I was not aware of a method companion

36. If the UK-HSA COVID-19 LA Reports were incorporated into the COVID-19 Situational Explorer dashboard, would this meet the needs of your organisation?

- Yes
- No

Additional indicators

37. Are there any additional indicators/information that could be provided to support decision making at this stage in the pandemic?

- Yes
- No

Alternative sources of information

38. What additional information sources do you use within your organisation to support decision making within the COVID-19 response? Please select all that apply

- UK-HSA dashboards
- Other UK-HSA reports
- Office for National Statistics (ONS)
- gov.uk
- gov.uk dashboard
- Care quality commission
- Academic publications
- Own information systems created within your organisation
- Other, please specify

Communication

39. Have you ever contacted the UK-HSA Outbreak Surveillance team for supplementary information or to provide feedback?

- Yes
- No

If answered "yes" to question 39:

40. Have you received the support required when contacting the UK-HSA Outbreak Surveillance team?*

- Yes
- No

Other

41. Do you have any additional suggestions/comments on how the UK-HSA Outbreak Surveillance team reports could be improved to support the work of your organisation?

42. Please enter your email address if you would be happy for us to contact you for additional feedback
